# Ex Vivo Evaluation of Ethosomes and Transethosomes Applied on Human Skin: A Comparative Study

**DOI:** 10.3390/ijms232315112

**Published:** 2022-12-01

**Authors:** Elisabetta Esposito, Laura Calderan, Andrea Galvan, Enrica Cappellozza, Markus Drechsler, Paolo Mariani, Alessia Pepe, Maddalena Sguizzato, Enrico Vigato, Edoardo Dalla Pozza, Manuela Malatesta

**Affiliations:** 1 Department of Chemical, Pharmaceutical and Agricultural Sciences, University of Ferrara, I-44121 Ferrara, Italy; 2 Department of Neurosciences, Biomedicine and Movement Sciences, University of Verona, I-37134 Verona, Italy; 3 Bavarian Polymer Institute (BPI), Keylab “Electron and Optical Microscopy”, University of Bayreuth, D-95440 Bayreuth, Germany; 4 Department of Life and Environmental Sciences, Università Politecnica delle Marche, I-60131 Ancona, Italy; 5 Department of Plastic and Reconstructive Surgery, Verona University Hospital (A.O.U.I. Verona), I-37126 Verona, Italy

**Keywords:** ethosomes, transethosomes, explanted skin, bioreactor, X-ray diffraction, cryogenic transmission electron microscopy, transmission electron microscopy

## Abstract

In this study, the transdermal fate of vesicular nanosystems was investigated. Particularly, ethosomes based on phosphatidylcholine 0.9% *w*/*w* and transethosomes based on phosphatidylcholine 0.9 or 2.7% *w*/*w* plus polysorbate 80 0.3% *w*/*w* as an edge activator were prepared and characterized. The vesicle mean size, morphology and deformability were influenced by both phosphatidylcholine and polysorbate 80. Indeed, the mean diameters of ethosome were around 200 nm, while transethosome’s mean diameters were 146 or 350 nm in the case of phosphatidylcholine 0.9 or 2.7%, *w*/*w*, respectively. The highest deformability was achieved by transethosomes based on phosphatidylcholine 0.9%, *w*/*w*. The three types of vesicular nanosystems were applied on explanted human skin maintained in a bioreactor. Transmission electron microscopy demonstrated that all vesicles were able to enter the skin, keeping their structural integrity. Notably, the vesicle penetration capability was influenced by their physical-chemical features. Indeed, ethosomes reached keratinocytes and even the dermis, phosphatidylcholine 0.9% transethosomes were found in keratinocytes and phosphatidylcholine 2.7% transethosomes were found only in corneocytes of the outer layer. These findings open interesting perspectives for a differentiated application of these vesicles for transdermal drug delivery as a function of the cutaneous pathology to be addressed.

## 1. Introduction

The skin is the outer organ, whose main role is protecting our body from external insults, acting as a barrier against environmental stressors and injuries [1]. Among the three layers composing the skin—epidermis, dermis and hypodermis—the major barrier function is played by the epidermis, with its outer layer constituting non-viable cells (i.e., stratum corneum) and the inner layers made of viable keratinocytes (i.e., stratum lucidum, stratum granulosum, stratum spinosum and stratum basale). On the other hand, the dermis is a greatly vascularized layer, making the skin a significant drug administration route. In particular, the topical route may be the best option to treat skin conditions and disorders [1,2]. Nevertheless, due to its peculiar structure, the stratum corneum layer acts as a barrier, hindering the passage of drugs through the skin [1]. To overcome this problem, many research efforts have been carried out to design efficacious transdermal delivery systems [3]. Particularly, colloidal-based delivery systems made of biocompatible and biodegradable phospholipids were developed. The former approach was based on the use of liposomes, bi-layered vesicles usually constituted of phospholipids, such as phosphatidylcholine (PC), and water, which is able to deliver both hydrophilic and hydrophobic molecules [3,4,5]. However, liposomes proved to not be flexible enough to penetrate the skin, forming a depot in the stratum corneum, from which the encapsulated drugs could slowly diffuse [6]. Therefore, deformable liposomes were developed using edge activators (e.g., surfactants) in order to destabilize the lipid bilayers formed by PC and water, possibly improving the flexibility of the vesicles [7,8]. More recently, a significant improvement of vesicle flexibility was achieved by Touitou’s invention of ethosomes (ET) [9,10,11]. ET composition is based on phospholipids (such as PC), water and an ethanol content comprised between 20 and 45% *v*/*v*. ET can solubilize both lipophilic and hydrophilic drugs inside the vesicles. The presence of ethanol confers elasticity to the vesicles, due to its ability to interact with both skin and vesicle lipids, promoting the passage of the entrapped drug through the stratum corneum. Moreover, ethanol improves the vesicle thermodynamic stability and loading capacity of lipophilic drugs in comparison to liposomes [11,12,13]. To further increase the vesicle penetration ability, transethosomes (TET) have been developed. TET are composed of phospholipids, such as PC, water, ethanol and edge activators, such as surfactants [13,14,15]. TET should therefore possess the advantages of both deformable liposome and ET, being constituted of both edge activators and ethanol, as well as PC and water [15]. Therefore, ET and TET can be described as flexible PC-based nano-vesicular systems that are suitable for achieving the transdermal delivery of many drugs, offering the chance to treat several skin pathologies [16,17,18,19,20,21,22,23,24,25].

In the development of a transdermal delivery system, the knowledge of its capability to maintain its structural integrity throughout the skin strata is of paramount importance in order to understand its efficacy as a drug carrier, especially when comparing different formulations.

Many studies have investigated the ET and TET transdermal effect in in vitro and in vivo models by evaluating the delivery of the entrapped drug through the skin and/or by demonstrating their therapeutic potential, as extensively reported in research articles and at least ten reviews [10,11,12,13,26,27,28,29,30,31]. Conversely, few studies were devoted to investigating the effective cutaneous penetration of ET and TET after skin administration [16,19]. To this aim, confocal scanning laser microscopy was usually employed, it being a technique that is suitable for detecting fluorescently labelled vesicles in human or animal tissues. For instance, a study evidenced the capability of TET to reach the lower epidermal region of rat skin [20], while a more recent investigation demonstrated the penetration of ET containing 5-fluorouracil through human hypertrophic scar tissue [21]. Particularly, the cutaneous penetration efficiency of ET fluorescently labelled with rhodamine 6GO was evaluated, mounting the skin between the donor and receptor phase of a diffusion cell. The images of skin cross-sections perpendicular to the skin surface showed a systematic increase in fluorescence intensity in the skin, indicating a time-dependent skin penetration of ET [21]. Other authors evaluated, by the same method, the fluorescent intensity of rhodamine 123 entrapped in ET, finding deshaped vesicles penetrated through the skin, suggesting the intact vesicle penetration through the stratum corneum. The fluorescence, found at different skin depths, was more intense in the case of ET with respect to hydroalcoholic solution [12]. Nevertheless, in both cases, the precise distribution of ET in various tissue layers was not detectable.

On the other hand, the uptake of ET and TET was evaluated by different authors in order to verify the vesicle capability to effectively cross the cell membrane [22,23,24]. Notably, a recent in vitro study described, at the ultrastructural level, the intracellular fate of ET and TET, revealing distinct degradation pathways for the two vesicle types [23].

To the best of our knowledge, the fate of ET and TET vesicles through the different human skin strata has never been investigated by transmission electron microscopy (TEM). To answer this unmet research need, in the present work, a comparative study was conducted, evaluating the ET and TET penetration capability and structural preservation throughout human skin. Briefly, in the first part of the study, ET and TET produced with different PC concentrations were characterised in order to verify their suitability for transdermal delivery; physico-chemical features such as morphology, size and deformability can in fact affect the passage through the skin [25,32]. In the second part of the study, the transdermal passage of ET and TET applied on human skin explants maintained in an innovative bioreactor for increasing times was analyzed by TEM. In vitro models of biological barriers mimicking in vivo physiological conditions are increasingly used in nanomedical research [33], and this experimental model proved to be a reliable tool to monitor transdermal delivery, since it ensures the optimal preservation of the skin explants structure, even for a long time under strictly controlled in vitro conditions [34]. In addition, thanks to its high resolution, TEM allows for the unequivocal identification of nanoconstructs, which can be visualized directly inside cells and tissues, without the need for markers, as it occurs at fluorescence microscopy [35], avoiding the underestimation of the signal in autofluorescent tissues such as the skin. Therefore, TEM was used as the most suitable tool for the detection of ET and TET inside the human skin.

## 2. Results

### 2.1. Preparation of Ethosomes and Transethosomes

ET and TET were designed and prepared to obtain biocompatible vehicles suitable for transdermal delivery. Both ET and TET were based on PC and ethanol, components characterized by penetration enhancer properties. The ET were constituted of PC 0.9% *w*/*w*, while the TET were obtained by enriching PC 0.9 (TET-1) or 2.7 (TET-2)% *w*/*w* with polysorbate 80 (T80) as the edge activator [14,15]. The low-energy-consuming method, based on a simple addition of water to the PC solution under stirring, enabled the rapid obtention of milky dispersions in the case of ET and translucent dispersions in the case of TET. Table 1 reported the compositions of ET and TET.

### 2.2. Characterization of Ethosomes and Transethosomes

#### 2.2.1. Size Distribution

A dynamic light scattering analysis was performed by Photon Correlation Spectroscopy (PCS) to study the effect of ET and TET composition on vesicle size distribution. Table 2 reports the Z Average vesicle mean diameters and dispersity indexes.

The Z-Average, between 146 and 350 nm, was strongly influenced by vesicle composition, both by PC concentration and T80 presence. Indeed, the mean size of ET vesicles, around 200 nm, was intermediate between the TET-1 and TET-2 vesicle sizes. In the case of TET-1, the presence of T80 led to a 60 nm decrease in the Z Average with respect to ET. Conversely, in the case of TET-2, the triple PC concentration (2.7%, *w*/*w*) led to a 1.7-fold increase in the Z-Average with respect to ET and a 2.4-fold increase with respect to TET-1. Dispersity index values were below 0.15 in the case of ET and TET-1, denoting a quite homogeneous size population. On the other hand, in the case of TET-2, the dispersity index was higher than 0.2, suggesting a broader size distribution [36]. 

**Figure 1:**
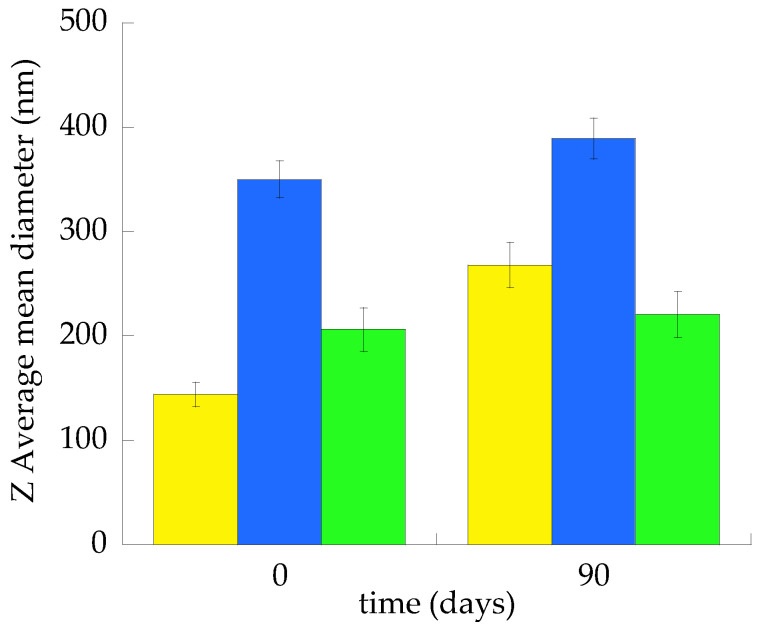
Mean diameters of ET (green), TET-1 (yellow) and TET-2 (blue), as measured by PCS at time 0 and after 3 months of storage at 22 °C. Data are the mean of three independent measurements ± s.d.

Figure 1 compares the Z Average mean diameters of the ET, TET-1 and TET-2 vesicles measured at time 0 and after 3 months of storage at 22 °C.

ET vesicles were more stable, displaying an almost 20 nm increase in the mean diameter in 3 months, followed by TET-2, undergoing a 40 nm increase. The major size increment was achieved in the case of TET-1, passing from 144 to 268 nm.

#### 2.2.2. Zeta Potential

The Zeta potential parameter gives an indication of colloidal system stability, since it reflects the degree of repulsion between the vesicles representing the disperse phase. The Zeta potential values of nanovesicular systems, as reported in Table 2, were generally above −20 mV, suggesting a high stability. The negative sign is related to the presence of ethanol, which provides a negative charge on the vesicle surface. The presence of T80 in TET-1 and TET-2 further increases the Zeta potential magnitude, while the higher PC amount in TET-2 scarcely affected the Zeta potential value [14].

#### 2.2.3. Morphology

**Figure 2:**
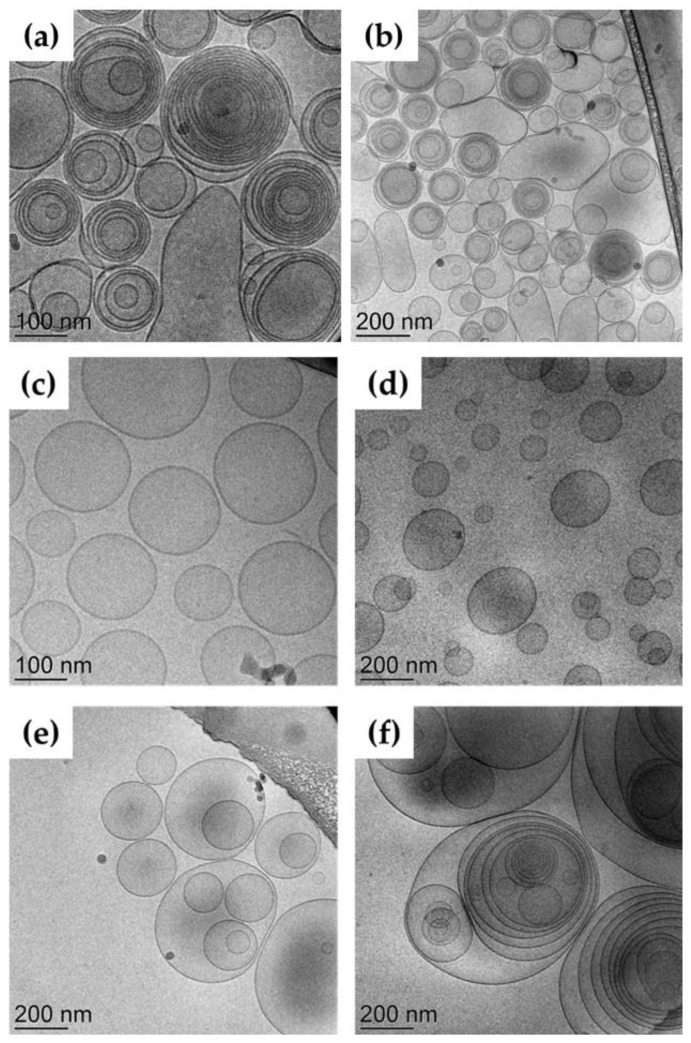
Cryo-TEM images of ET (**a**,**b**) TET-1 (**c**,**d**) and TET-2 (**e**,**f**).

The ET and TET morphology visualized by cryogenic transmission electron microscopy (cryo-TEM) shed light on the effect of composition on the supramolecular architecture of vesicles. Figure 2 reports some micrographs of the different nanocarriers.

ET vesicles (panels a and b) appeared mainly spherical and multilamellar, apart from some larger unilamellar structures. A completely different organization was found in the case of TET-1 (panels c and d), showing unilamellar spherical vesicles. Conversely, TET-2 was characterized by multilamellar and multivesicular vesicles.

**Figure 3:**
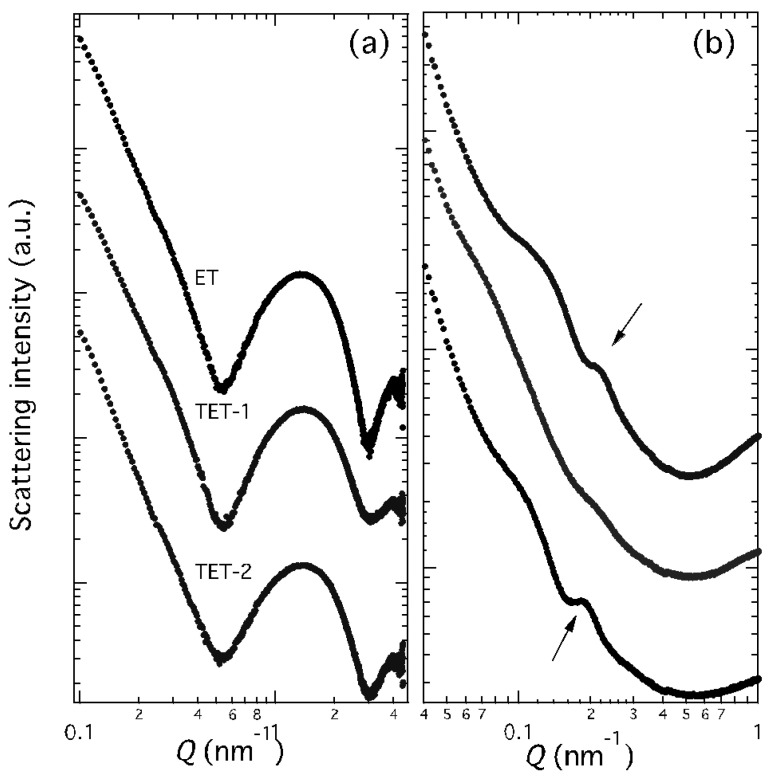
SAXS profiles observed at 35 °C for ET, TET-1 and TET-2 vesicles, as indicated. Panels (**a**) and (**b**) only differ for the investigated *Q* range. Arrows in panel (**b**) indicate the main diffraction peaks.

The inner morphological organization of ET, TET-1 and TET-2 was investigated by X-ray scattering. The SAXS profiles are reported in Figure 3. As panel (a) shows, typical bilayer form factor scattering patterns (e.g., the broad band centred at about 1.5 nm^−1^) are observed in the three different samples [22,37]. Two points should be noticed: (i) the profiles denote the presence of PC double-layered vesicles at all the investigated conditions; (ii) the broad band position was independent of the vesicle composition, confirming the relationship with a bilayer electron density profile, which remains similar. Accordingly, the bilayer thickness was measured as approximately 4.8 nm [38].

However, in very good agreement with the cryo-TEM images, the X-ray scattering patterns of ET and TET-2 show the presence of low-intensity Bragg peaks superposed to the bilayer form factor (visible in panel (b) of Figure 3). In both cases, only one peak was observed, as a clear indication of the formation of PC multilamellar vesicles characterized by a very disordered positional correlation between adjacent bilayers and/or by a very limited number of closed bilayers [38,39]. The positional correlation distance between adjacent bilayers was larger in TET-2 (around 33 nm) than in the ET vesicles (around 29 nm).

Therefore, SAXS data confirmed that both PC concentration and the presence of T80 affect the vesicle structures, even if the PC organization in double-layered lamellae is maintained, regardless of the vesicle composition. 

### 2.3. Deformability Study

The deformability of ET and TET represents a peculiar feature that can influence their capability to pass through natural membranes such as the skin [32]. To compare the effect of PC concentration and T80 on the deformability of vesicles, the variation in mean diameter was evaluated under vesicle extrusion. The results are summarized in Table 2. TET-1 was 1.53× more elastic than ET, while TET-2’s deformability was the lowest. As suggested by SAXS and cryo-TEM measurements, deformability can be correlated with vesicle structures: in TET-1, the presence of T80 promotes the formation of unilamellar vesicles, increasing the vesicle deformability; in TET-2, multilamellarity and, more importantly, the presence of multivesicular vesicles induce rigidity.

### 2.4. Transmission Electron Microscopy Study of Skin Penetration

ET, TET-1 and TET-2 were applied ex vivo on human skin in order to visualize their passage through the different epithelial strata. Explanted human skin samples were maintained in a bioreactor under fluid dynamic conditions [34], thus mimicking the physiological state and improving structural and functional preservation. The vesicles were applied on the stratum corneum and then incubated for 1 h, 3 h, 6 h and 24 h. At each incubation time, skin samples were fixed and processed for TEM. In parallel, skin samples maintained under the same experimental conditions but treated with the suspension medium devoid of vesicles were used as control.

**Figure 4:**
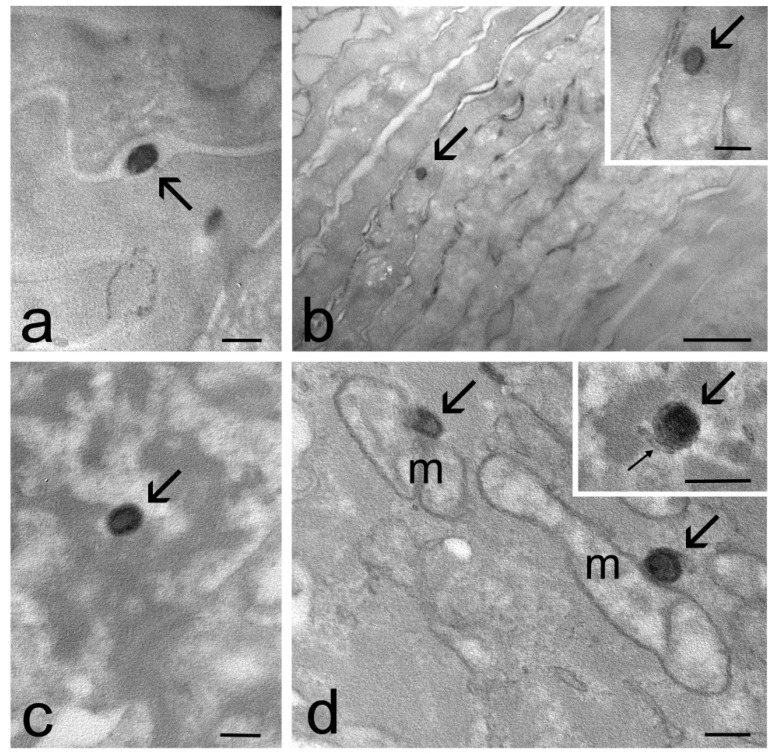

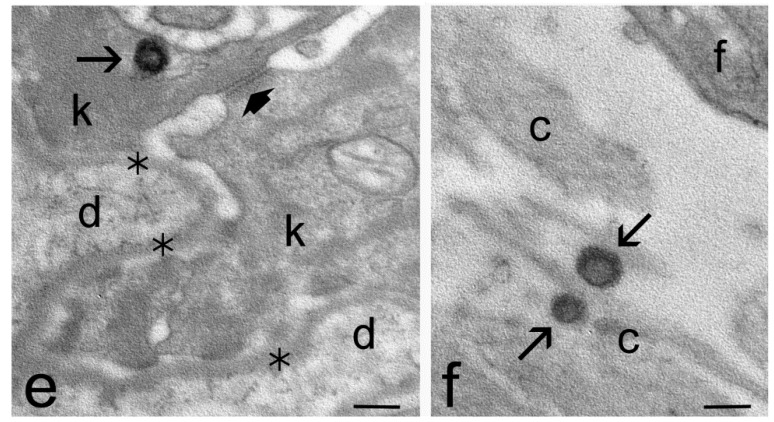
TEM micrographs of ET in the skin. (**a**) An ET (arrow) occurs in the intracellular space of the stratum corneum. (**b**) An ET (arrows) has been internalized in a corneocyte. In the high-magnification micrograph, the dark rim and the weakly electron-dense core of the ET (arrow) are clearly visible. (**c**) An ET (arrow) in the cytoplasm of a keratinocyte belonging to the stratum granulosum. (**d**) ET (arrows) make contact with mitochondria (m) and smooth endoplasmic reticulum cisternae (thin arrow in the inset). (**e**) An ET in the cytoplasm of a keratinocyte (k) of the stratum basale. Note the well-preserved desmosome (arrowhead) and the basal lamina (asterisks) connecting keratinocytes (k) to the dermis (d). (**f**) Two ET (arrows) in the extracellular matrix of the upper papillary dermis; c, collagen bundles; f, fibroblast. Bars: 200 nm (**a**,**c**–**f**, inset **b**); 1 µm (**b**).

**Figure 5:**
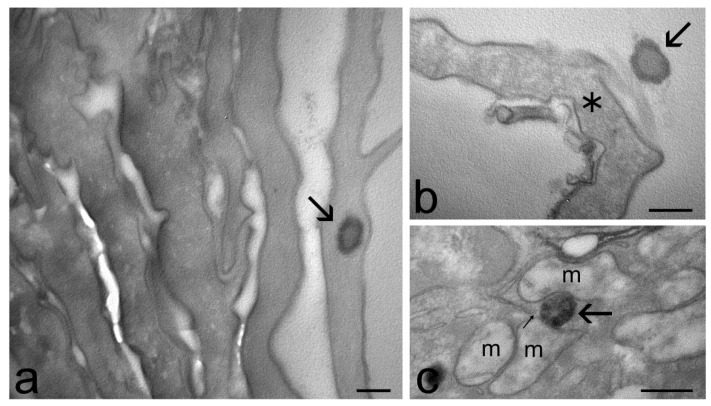
TEM micrographs of TET-1 in the skin. (**a**) A TET-1 (arrow) has been internalized into a corneocyte. (**b**) A TET-1 (arrow) occurs in the intercellular space of the stratum corneum, close to a corneocyte (asterisk). (**c**) A partially degraded TET-1 (arrow) inside a keratinocyte is located very close to smooth endoplasmic reticulum cisternae (thin arrow) and mitochondria (m). Bars: 200 nm.

**Figure 6:**
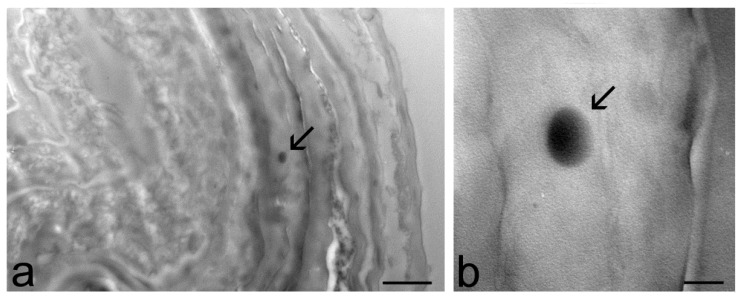
TEM micrographs of a TET-2 (arrows) inside a corneocyte of the outer layer of the skin (**a**). (**b**) High-magnification detail: note the homogeneous dark content of the vesicle. Bars: 1 µm (**a**); 200 nm (**b**).

At TEM, ET and TET were clearly recognizable inside the skin as roundish, strongly electron-dense structures (Figure 4, Figure 5 and Figure 6). In particular, ET and TET-1 showed a darker rim, as previously reported [23], whereas TET-2 showed a homogeneous dark content. The mean diameter ± s.d. of vesicles measured on TEM images was 193.38 ± 20.77 nm for ET, 151.72 ± 17.29 nm for TET-1 and 274.35 ± 41.18 nm for TET-2.

After 1 h and 3 h of incubation, many ET were observed in the stratum corneum, both inside corneocytes and in the interstices among them (Figure 4a,b), while they occurred in smaller amounts inside keratinocytes, with a gradually dropping frequency from the upper to the basal strata (Figure 4c–e). After 3 h of incubation, rare ET were found in the connective tissue of the upper papillary dermis that underlies the epidermis (Figure 4f).

Many TET-1 were found after 1 h both in the intercellular space and inside the cells of the stratum corneum (Figure 5a,b), but their presence became less frequent after 3 h of incubation. Moreover, TET-1 were found in a low amount inside keratinocytes (Figure 5c) and were never observed in the dermis. Both ET and TET-1 were observed making contact with smooth endoplasmic reticulum and mitochondria inside keratinocytes (Figure 4d and Figure 5c).

TET-2 were rarely found only in the stratum corneum after 1 h or 3 h of incubation (Figure 6).

After 6 h and 24 h, no ET, TET-1 or TET-2 was ever found.

No sign of structural alteration due to vesicle application was observed in any skin components in comparison to controls (not shown). 

## 3. Discussion

The possibility of administering drugs through the skin enables the treatment of many cutaneous pathologies and disorders. Research efforts led to the development of different approaches, including the administration of colloidal vesicular systems. Nonetheless, since the fate of these vesicular systems upon cutaneous administration represents an open question, in the present investigation, a new ex vivo approach is described for detecting the transdermal potential of vesicles applied on the human skin. The first part of the research was devoted to the design and characterization of ET and TET based on PC, ethanol, water and the surfactant T80. The choice of PC concentration, as well as the type and amount of the surfactant, was taken based on previous findings about mangiferin-loaded vesicles [14,40]. Particularly, a formulative study was conducted, evaluating the effect of PC and surfactants on vesicle size distribution and stability. PC 0.9% was selected for ET preparation, it being suitable for obtaining a small mean diameter and maintaining the vesicle size stability for a long time, whilst higher PC concentrations led to sedimentation and phase separation of the disperse phase. Conversely, in the case of TET, T80 0.3% was employed in a mixture with PC 0.9 or 2.7%, allowing for the stabilization of even the dispersions based on the highest amount of PC, preventing phase separation [40]. Accordingly, in the present investigation, three kinds of vesicular systems were chosen. It was confirmed that both PC concentration and the presence of T80 strongly affected the vesicle structures. Indeed, it is well known that the peculiar structure of PC, constituting two hydrophobic tails and one hydrophilic head group, results in the spontaneous formation of double-layered vesicles that organize in a multilamellar concentric structure in the case of ET [14]. T80 was previously selected by a formulative study as a surfactant suitable for controlling both the size distribution and stability of vesicles [14,23]. It possesses an oleate chain that is thought to intercalate within the PC bilayer of TET vesicles, hampering the formation of multilamellar vesicles in the case of low concentrations of PC (0.9%, *w*/*w*) [14,23]. Conversely, the same amount of T80 in the presence of three-fold-higher concentrated PC (2.7%, *w*/*w*) led to multilamellar and oligovesicular vesicles [24]. Consequently, PC concentration has a crucial effect on the vesicle mean diameters, which are larger in the case of multilamellar and oligolamellar vesicles with respect to unilamellar ones, as observed by PCS and cryo-TEM. Notably, the enlargement of vesicle mean diameters under an increase in PC concentration was previously observed by different authors [41,42].

The different lamellar organization of ET, TET-1 and TET-2 could explain the different appearances of the three types of vesicles observed by TEM in the skin. In fact, ET and TET-1, characterized by the presence of one lamella or many concentric lamellae, showed a peripheral darker rim, likely due to the electron density of osmiophylic lipid lamellae [35], and a less electron-dense core, where lamellae are absent. On the other hand, TET-2 was homogeneously electron-dense, probably due to the heterogeneous distribution of osmiophylic lamellae in its interior. 

Remarkably, the roundish shape of ET and TET observed inside the skin by TEM suggests that the vesicles preserve their structural integrity while passing through the epidermis. Moreover, the sizes of the ET and TET internalized in the skin were similar to the PCS data, demonstrating that the penetration into the skin does not affect vesicle size. However, only a few TET-2 were found in the skin, located exclusively in the upper layer of the stratum corneum, suggesting that their scarce deformability and large size may hamper their penetration capability. Reasonably, vesicles with a smaller size have a better chance of penetrating through the skin compared to the larger ones; indeed, the size of lipid vesicles strongly influences their penetration through biological membranes, affecting the bioactive delivery to the skin, as previously demonstrated [24,36]. Conversely, ET and TET-1, characterized by a lower size and higher deformability, were found in different epithelial strata, and ET was found even in the dermis (Table 3). 

Remarkably, both ET and TET-1 entered the skin rapidly and were found in the epidermis after 1 h of incubation; nevertheless after 6 h of incubation, no more vesicles were observed, suggesting their complete degradation. Previous findings in cultured cells (included keratinocytes) [23] demonstrated that both ET and TET-1, mostly made of PC, were massively degraded by smooth endoplasmic reticulum-resident enzymes (likely phospholipase D) [43]. Moreover, PC molecules were translocated to mitochondria to be assembled into their membranes [44]. However, in vitro, this process required short times (1–2 h) for TET-1, whereas ET were still morphologically recognizable after 24 h. This different sensitivity to phospholipases could be due to the presence of T80, which affects the molecular packing of PC in the TET-1 vesicle layers, making them more degradable. In explanted human skin, the degradation time of both ET and TET-1 was more rapid than that in cultured cells; indeed, no vesicle was still detectable after 6 h of incubation, but TET-1 confirmed their faster degradability, as shown by their decreased presence after 3 h of incubation. 

As demonstrated by TEM observations, ET, TET-1 and, to a much lesser extent, TET-2 do penetrate almost intact into the stratum corneum by slipping in the interstices between corneocytes. It is likely that ET and TET are able to move in the lipid matrix thanks to their chemical and structural similarity with the skin lipids [45]. In addition, ET and TET are characterized by a malleable structure that facilitates their movement in the intracellular interstices, and the presence of ethanol acts as a permeation enhancer, inducing the disorganization of the lamellar lipid-enriched matrix embedding corneocytes [46]. Notably, ET and TET proved to also be able to enter the corneocytes. Previous studies [23] demonstrated that ET and TET are able to pass through the plasma membrane, maintaining their structural integrity without an endocytic process. Based on the chemical and structural similarities between ET/TET and plasma membrane (PC is a major phospholipid component of biological membranes [47]), it has been hypothesized that a fusion with the plasma membrane occurs [48,49]. This phenomenon could take place thanks to the presence of ethanol, which would induce the loosening of lipid packaging in the plasmalemma region making contact with the vesicles [50]. Interestingly, these malleable vesicles proved to be able to cross the cell membrane of corneocytes, which is reinforced by a cross-linked protein layer at the cell periphery [51]. 

Impressively, ET and TET-1 were found inside keratinocytes, from the stratum spinosum until the stratum basale (with a progressively decreasing degree), even though the mechanism(s) of their penetration remains unclear. Keratinocytes are strictly connected each other by numerous desmosomes, which represent an efficient barrier that is very difficult to cross. It is worth noting that the innovative bioreactor used in this work is especially suitable for preserving the structural and functional features of explanted skin, even for a long time [34], and TEM observations clearly demonstrated the occurrence of typical desmosomes in the samples incubated with ET and TET. It seems therefore unlikely that ET and TET-1, despite their high deformability, could be able to move in the extracellular matrix of the epidermal layers under the stratum corneum. Accordingly, we never found ET or TET among the keratinocytes, but only inside them. This supports the hypothesis that the penetration into the skin (especially the lower epidermal layers) takes place through the transcellular way. This hypothesis would also fit the higher amount of ET found in the lower keratinocyte strata compared to TET-1, which undergo a more rapid intracellular degradation [23]. The greater resistance of ET to enzymatic degradation would also explain why so few of them were found, even in the upper papillary dermis. Interestingly, this finding reveals the capability of ET to cross both the keratinocyte plasma membrane and basal lamina.

These findings strongly confirm the potential of ET and TET-1 as transdermal delivery systems that are able to pass through the stratum corneum barrier while maintaining their structure for at least 1 h after their administration. Remarkably, ET and TET-1 uptake in keratinocytes suggests the possibility of delivering the loaded drug directly inside the cells. Notably, the different composition and ultrastructure of ET and TET-1 could account for their diverse degradation times. Indeed, in the case of ET, the PC multilamellar vesicles were detectable for longer with respect to TET-1. In this latest case, T80/PC unilamellar vesicles could possibly release the loaded drug faster than ET.

Finally, the treatment with ET or TET was demonstrated to be well tolerated by the skin, since no structural alteration was observed in the cellular and extracellular components of the epidermis and dermis of the explanted human skin samples. This evidence agrees with a previous in vitro and in vivo studies on ET [52], as well as with in vivo studies conducted on health volunteers to evaluate the possible irritant reactions induced by the cutaneous application of ET, TET-1 and TET-2 [24,53]. The results indicated that ET, TET-1 and TET-2 can be classified as not irritating if applied to human skin, confirming the suitability of these nanosystems for cutaneous administration.

## 4. Materials and Methods

### 4.1. Materials

The soybean lecithin (PC) (92% PC) was Epikuron 200 from Lucas Meyer (Hamburg, Germany). Polyoxyethylenesorbitan monooleate and T80 were purchased from Sigma-Aldrich (St Louis, MO, USA). The solvents were of HPLC grade; all other chemicals were of analytical grade.

### 4.2. Ethosome and Transethosome Preparation

ET and TET were produced by the “cold method” [14,54,55,56]. Particularly, PC was previously solubilized in ethanol (30 or 90 mg/mL) under stirring at 750 rpm (IKA RCT basic, IKA^®^-Werke GmbH & Co. KG, Staufen, Germany); afterwards, bi-distilled water was added dropwise up to a final 70:30 (*v*/*v*) ratio under magnetic stirring at 750 rpm. The stirring was then maintained for 30 min at 22–25 °C [14]. TET preparation was similarly obtained upon the previous addition of T80 1% *w*/*v* to the PC solution before the addition of water. Table 1 reports ET and TET compositions. 

### 4.3. Photon Correlation Spectroscopy and Zeta Potential Measurements

Zetasizer Nano-S90 (Malvern Instr., Malvern, England), with a 5 mW helium neon laser and a wavelength output of 633 nm, was employed for the vesicle size analysis of ET and TET. Measurements were performed at 25 °C at a 90° angle and a run time of at least 180 s, diluting samples with bi-distilled water in a 1:20 *v*/*v* ratio. Data were analyzed using the “CONTIN” method [57]. Measurements were performed thrice for 3 months from ET and TET production. Zeta potential values were acquired by measuring the electrophoretic mobility according to the Hemholtz–Smoluchowski equation [58].

### 4.4. Cryo-Transmission Electron Microscopy

Samples for cryo-TEM were vitrified by putting sample droplets (2 µL) for some seconds on a lacey-carbon-filmed copper grid (Science Services, Munich, Germany) [14]. Afterwards, most of the liquid was removed by blotting paper, obtaining a thin film stretched over the lace holes. The rapid immersion of the specimen into liquid ethane cooled to approximately 90 K by liquid nitrogen in a temperature-controlled freezing unit (Leica EMGP, Leica, Germany) instantly allowed for their vitrification. All sample preparation steps were conducted at a controlled constant temperature in the Leica EMGP chamber. The vitrified specimen was transferred to a Zeiss/Leo EM922 Omega EFTEM (Zeiss Microscopy GmbH, Jena, Germany) transmission electron microscope using a cryoholder (CT3500, Gatan, Munich, Germany). During the microscopy observations, the sample temperature was kept below 100 K. Specimens were examined with reduced doses ≈ 1000–2000 e/nm^2^ at 200 kV. Zero-loss filtered images (∆E = 0 eV) were recorded by a CCD digital camera (Ultrascan 1000, Gatan, Munich, Germany) and analyzed using GMS 1.9 software (Gatan, Munich, Germany).

### 4.5. X-Ray Scattering

X-ray scattering experiments were performed at the bioSAXS beamline B21 of the Diamond Light Source (Harwell, UK). Samples were placed into PCR tubes in an automated sample changer and then transferred to a temperature-controlled quartz capillary and exposed for 1 s, acquiring 30 frames at 35 °C in order to check equilibrium conditions and to eventually monitor radiation damage. Data collection was performed using a Pilatus Dectris 2 M detector. The X-ray wavelength λ was 0.10 nm, and the explored *Q*-range (*Q* is the modulus of the scattering vector, defined as 4π sin θ/λ, where 2θ is the scattering angle) was 0.1 to 4.4 nm^−1^. Two-dimensional data were corrected for the background, detector efficiency and sample transmission and then radially averaged to derive I(*Q*) vs. *Q* curves [59]. A few experiments were performed at a lower *Q*-range, exploring the region from 0.04 to 1.0 nm^−1^.

### 4.6. Deformability Measurement

The deformability of nanovesicular systems was determined by an extrusion method [32]. ET and TET were extruded through a polycarbonate filter membrane (pore diameter 50 nm) using a stainless steel, 25 mm diameter filter holder (extruder) and applying a 2.5 bar pressure at 25 °C. The volume of ET and TET formulation extruded in 1 min was measured. Vesicle size was measured by PCS before and after the extrusion. The deformability of the vesicle membrane was calculated according to the following equation:

Def = J × (rv/rp)^2^ (1)

where Def is the vesicle deformability; J is the ratio between the volume of extruded formulation (mL) and the time of extrusion (min); rv is the vesicle size expressed in nm (after extrusion); and rp is the pore size of the filter membrane.

### 4.7. Transmission Electron Microscopy Study of Skin Penetration

Healthy skin samples were obtained, after informed consent, from the abdominal or breast region of four healthy women (24–45 years old) undergoing plastic surgery. Freshly excised skin samples were rapidly washed in physiological solution (NaCl 0.9% *w*/*v*) and then in pre-warmed (37 °C) culture medium containing DMEM (Dulbecco’s Modified Eagle’s Medium), 4.5 g/L D-glucose, 10% FBS, 2% penicillin–streptomycin, 200 mM L-glutamine and 0.3 μg/mL Amphotericin B (Gibco, Waltham, MA, USA). Roundish samples that were 1.5 cm in diameter were placed in a bioreactor (IV-Tech, Massarosa, LU, Italy) and mounted in culture chambers (modified LiveBox2) with the epidermis facing up, in contact with air, and the dermis facing the lower side, in contact with the flowing culture medium, as described in [34]. A flow rate of 500 μL/ min was applied to the culture medium. A 300 µL drop of ET, TET-1 or TET-2 suspension (PC 0.9% *w*/*w* for ET and TET-1, 2.7% *w*/*w* for TET-2) was applied on the surface of the skin from the upper route of administration of the culture chamber immediately after mounting and locking the explants into the bioreactor [60]. Then, the bioreactor was placed in the incubator, at 37 °C in 5% CO_2_ humidified atmosphere, for 1 h, 3 h, 6 h and 24 h. Some samples were incubated with the suspension medium of vesicles as controls.

At the end of each incubation time, skin samples were fixed with 2% (*v*/*v*) paraformaldehyde and 2.5% (*v*/*v*) glutaraldehyde in 0.1 M phosphate buffer at 4 °C for 3 h, post-fixed with 1% (*v*/*v*) OsO_4_ and 1.5% (*v*/*v*) potassium ferrocyanide for 1.5 h at room temperature, dehydrated with acetone and embedded in Epon-Araldite mixture. Ultrathin sections (70–90 nm thick) were stained with Reynold’s lead citrate and observed with a Philips Morgagni transmission electron microscope (FEI Company Italia Srl) operating at 80 kV and equipped with a Megaview II (Olympus SIS, now EMSIS, Münster, Germany) camera for digital image acquisition.

ET, TET-1 and TET-2 diameters were measured in TEM micrographs (×22,000) using ImageJ software (NIH), and the mean values ± s.d. were calculated (n = 25 for ET and TET-1; n = 7 for TET-2). Although these values cannot be compared with those obtained with PCS, since the samples observed at TEM underwent fixation, dehydration, embedding and cutting, in our experience, TEM and PCS measurements generally give similar results. TEM measurements therefore represent a reliable technique for obtaining information on the possible effects of the biological environment on the vesicle size.

## 5. Conclusions

In this study, different formulations of ET and TET were prepared, characterized for their physico-chemical properties and applied to explanted human skin maintained in an innovative bioreactor. The high resolution of TEM, which allows for the direct visualization of vesicles and tissue components without recourse to markers, unequivocally demonstrated their penetration capability and their fate inside the various skin layers.

To enter the skin, ET and TET must have an appropriate size and deformability features. The vesicles that are able to penetrate the epidermal barrier (ET and TET-1) crossed the stratum corneum through the intercellular space but also entered corneocytes, whereas, from the stratum granulosum to the stratum basale, the passage probably occurs transcellularly. In the intracellular environment, both ET and TET-1 undergo enzymatic degradation, and their resistance to this process determines the depth of the skin penetration (the more degradable it is, the less it penetrates). 

This information appears particularly crucial in achieving efficacious transdermal drug delivery approaches as a function of the cutaneous pathology to be addressed. Specifically, TET-1 could be proposed in the case of cutaneous diseases affecting more superficial strata, such as acute inflammatory dermatoses, and ET could be proposed to treat deeper and more serious conditions, such as basal cell carcinoma and melanoma.

## Figures and Tables

**Table 1:** Composition of nanovesicular systems (*w*/*w*%).

Components	ET	TET-1	TET-2
PC ^1^	0.90	0.89	2.69
T80 ^2^	-	0.30	0.30
Ethanol	29.10	28.81	27.01
Water	70.00	70.00	70.00

^1^ phosphatidylcholine; ^2^ polysorbate 80.

**Table 2:** Size distribution parameters, zeta potential and deformability of nanovesicular systems.

Parameters	ET	TET-1	TET-2
Z-Average (nm) ^1^ ± s.d.	206.32 ± 33.22	146.21 ± 10.21	350.40 ± 23.61
Dispersity index ^1^ ± s.d.	0.14 ± 0.00	0.13 ± 0.00	0.23 ± 0.04
Zeta potential (mV) ± s.d.	−23.39 ± 0.20	−33.65 ± 0.30	−35.01 ± 0.71
Def (mL/min) ^2^ ± s.d.	6.23 ± 0.71	9.55 ± 0.52	5.74 ± 0.52

^1^ as determined by PCS; ^2^ vesicle deformability; s.d.: standard deviation; data are the mean of three independent determinations on different batches.

**Table 3:** Distribution of vesicular nanosystems through the skin, as determined ex vivo by TEM.

Skin Strata	ET	TET-1	TET-2
Stratum corneum	yes	yes	yes
Keratinocytes	yes	yes	no
Dermis	yes	no	no

## Data Availability

Data are contained within the article. Additional data are available from the corresponding author upon reasonable request.
